# The association of medical, social, and normative factors with the implementation of end-of-life care practices

**DOI:** 10.1186/s13584-024-00589-w

**Published:** 2024-01-09

**Authors:** Arnona Ziv, Adir Shaulov, Carmit Rubin, Bernice Oberman, Yoel Tawil, Giora Kaplan, Baruch Velan, Moran Bodas

**Affiliations:** 1 The Gertner Institute for Epidemiology and Health Policy Research, Sheba Medical Center, Tel-Hashomer, Israel; 2 Department of Hematology, Hadassah Medical Center, Faculty of Medicine, Hebrew University of Jerusalem, Jerusalem, Israel; 3 Department of Emergency and Disaster Management, School of Public Health, Faculty of Medicine, Tel-Aviv University, PO Box 39040, 6997801 Tel-Aviv-Yafo, Israel

**Keywords:** End-of-life care, Palliative care, Family caregivers, Socio-normative attitudes

## Abstract

**Background:**

End-of-life (EoL) care practices (EoLCP) are procedures carried out at the EoL and bear directly on this stage in the patient’s life. Public support of these practices in Israel is far from uniform. Previous studies show that while ∼30% of participants support artificial respiration or feeding of terminally ill patients, 66% support analgesic treatment, even at the risk of shortening life. This study aimed to create a typology of six end-of-life care practices in Israel and assess the association of medical, social, and normative factors with the implementation of those practices. These practices included mechanical ventilation, artificial feeding, deep sedation, providing information to the patient and family caregivers, including family caregivers in EoL decision-making, and opting for death at home.

**Methods:**

This cross-sectional study was performed as an online survey of 605 adults aged 50 or more in Israel, of which ~ 50% (*n* = 297) reported supporting a dying terminally ill relative in the last 3 years. Participants were requested to provide their account of the EoL process of their relative dying from a terminal illness in several aspects, as well as the EoL care practices utilized by them.

**Results:**

The accounts of the 297 interviewees who supported a dying relative reveal a varied EoL typology. The utilization of end-of-life care practices was associated with the socio-normative beliefs of family caregivers but not with their socioeconomic status. Strong correlations were found between family caregiver support for three key practices (mechanical ventilation, artificial feeding, and family involvement in EoL) and the actual utilization of these practices in the care of dying patients.

**Conclusions:**

The findings portray an important image of equity in the utilization of EoLCP in Israel, as the use of these practices was not associated with socioeconomic status. At the same time, the study found substantial diversity in family caregivers’ preferences regarding EoL care practices use not related to socioeconomic status. We believe that differences in preferences that do not lead to problems with equity or other important societal values should be respected. Accordingly, policymakers and health system leaders should resist calls for legislation that would impose uniform EoL practices for all Israelis. Instead, they should take concrete steps to preserve and enhance the widespread current practice of practitioners to adapt EoL care to the varied needs and preferences of Israeli families and cultural, social, and religious subgroups. These steps should include providing frameworks and tools for family caregivers to support their loved ones close to their deaths, such as educational programs, seminars, supportive care before and during the end of life of their loved ones, etc.

## Background

According to the International Association for Hospice and Palliative Care (IAHPC), Palliative Care is defined as *“the active holistic care of individuals across all ages with serious health-related suffering due to severe illness and especially of those near the end-of-life.”* [1] Palliative care strives to improve the quality of life of patients and their family caregivers. Included within palliative care is End-of-Life care, which is provided in the last few weeks of the patient’s life [2].

The period before death entails, by its nature, close interactions between patients and the health care system. This is manifested, among others, by utilizing an extensive repertoire of healthcare services. Most of these services are part of routine medical practice, yet some are closely related to dying. End-of-life care practices (EoLCP) can be defined as procedures carried out at the end-of-life and bear directly on this stage in the patient’s life [1].

End-of-life care practices include procedures that are predominantly medical, such as managing symptoms, resuscitation, pain relief, and sedation, but also those that entail modifying the patient’s environment to ensure privacy, providing information to patients and their family caregivers, and enhancing confidence in the medical teams [3–7].

In this study, we focused on implementing six diverse end-of-life care practices: Three are medical, namely, mechanical ventilation, artificial feeding, and deep sedation. Another three are related to modulating the care environment: providing information, including family caregivers in decision-making, and opting for death at home.

In a recent systematic literature review, González-González et al. [8] looked into the synthesized data of 22 studies. The authors report that a fifth of patients (21%) in four studies preferred any life-sustaining treatment solution. Other studies reported lower rates and a general trend of declining such treatment when patients were closer to their deaths. In 13 studies, an average of 48% of patients were willing to be connected to mechanical ventilation. In three studies, on average, 52% of patients wished to die at home. Similar findings were reported in an earlier systematic review of end-of-life care practice utilization. For example, studies report that about half of patients are connected to mechanical ventilation at the end of life [9]. A recent study evaluating these practices in a tertiary public hospital in Brazil reported rates of enteral (artificial) feeding ranging from 39 to 57% and prescribed Morphine in ranges of 36–44%. Other studies report lower rates of end-of-life care practice utilization [10].

To date, there is no account for the typology of the utilization of end-of-life care practices in Israel. Previous studies primarily assessed the attitudes and opinions of Israeli physicians [11] and the general public [12]. More prominently, there is no account for the effect of caregivers’ socio-normative attitudes on the utilization of end-of-life care practices.

The current study focuses on the following end-of-life care practices: mechanical ventilation, artificial/enteral feeding, pain management with analgesics that could shorten lifespan, preparation, and utilization of advanced health directives, truth-telling about impending death, and the involvement of family caregivers in EoL decisions. The utilization of each and all of these end-of-life care practices should follow certain principles, namely being medically appropriate per the patient’s status, respecting the patient’s autonomy, and just using these practices without prejudice. Therefore, the purpose of this study was to provide a typology of end-of-life care practices utilization in Israel and its associated factors and account for the extent to which the utilization of these practices in Israel adheres to the principles mentioned above.

## Methods and materials

### Study type and design

This cross-sectional study was performed in late March 2022.

### Population and sampling

Included in this study were adults aged 50 or more in Israel. The choice to limit the minimum age to 50 was taken to increase the proportion of people who will report supporting a dying relative from a terminal illness in the past 3 years. According to the Israeli Central Bureau of Statistics, this group includes roughly 30% of the population (~ 2.8 million) [13].

The minimum sample size for a representative sample of this age group, with a 95% level of confidence, a maximal marginal error of 5%, and an expected frequency of one of the key EoL attitudes assessed (70% support of truth-telling [12]) was 323, according to OpenEpi Sample Size Calculator [14]. Participant recruiting and data collection were conducted using *iPanel*, an online polling service. Since 2006, iPanel has provided an online platform for various information collection services, including polls and public opinion surveys. The panel adheres to the stringent standards of the World Association for Market, Social, and Opinion Researchers (the European Society for Opinion and Marketing Research, ESOMAR). Random sampling was performed from a pool of more than 150,000 iPanel panelists with quotas for gender, age (50 and above), religion (Jewish/other), and geographical distribution. Variations in education, household income, and religiosity were also obtained (see Table 1). The final sample comprised 605 participants who agreed to participate, of which nearly 50% (*n* = 297) reported supporting a dying relative suffering from a terminal illness who died during the last 3 years.

### Variables and tools

Participants were requested to provide their account of the end-of-life process of their relative dying from a terminal illness in several aspects using a 64-item closed structured questionnaire. These included a nominal description of the protagonist patient and their EoL process (relationship status to the caregiver (first-degree or second-degree family member, close friend, other), types of major morbidities (e.g., malignancy, heart condition, stroke, diabetes, dementia), length of being dependent on nursing care, place of death (home, hospital, hospice, geriatric institution, other), length of the dying process, age at death, and the cause of death.

In addition, accounts were also made concerning end-of-life care practices administered to the dying relative using binary responses (yes/no), with alternative options of “do not remember,” “cannot decide,” or “irrelevant,” including the decision to stop life-prolonging treatment, mechanical ventilation, artificial feeding, use of strong analgesics (i.e., opiates), disclosing of approaching death to the patient, respect of the dying patient’s end-of-life care wishes (e.g., advanced directives), and the involvement of the family caregivers in EoL decisions (e.g., disclosing of approaching death, discussions of care options, participation in treatment choices/decisions).

The questionnaire also assessed different EoL-related normative attitudes and beliefs on a four-point Likert scale (not at all, somewhat, much, and very much) pertaining to the end-of-life care practices covered in the study, including truth-telling (e.g., disclosing approaching death), medically assisted dying, utilization of these practices (mechanical ventilation, artificial feeding, use of strong analgesics (i.e., opiates), allowing death at home, stopping life-prolonging treatment, advanced directives, and family caregivers’ engagement in end-of-life care. Socio-normative attitudes were evaluated prior to the assessment of actual end-of-life care practices.

Items pertaining to socio-normative attitudes toward end-of-life care practices, including truth-telling and disclosing approaching death to dying patients and their caregivers, stopping life-prolonging treatment, using analgesics that may shorten life, respecting advanced directives, and involvement of family caregivers in EoL decisions were tested for validity using the Cronbach’s alpha test. These items scored a Cronbach’s alpha value of 0.71. The tool can be made available upon reasonable request.

### Statistical analysis

Descriptive statistics regarding categorical demographic and background parameters (i.e., gender, place of birth, religion and religiosity, family status, household income, education, profession, political affiliation, and personal experience supporting a terminally ill relative over the past 3 years) are presented as numbers and percentiles. Continuous variables (i.e., age and number of members in the household) are described as mean and standard deviation.

All questions of interest were compared between age groups, religious affiliation, and political affiliation. Categorical variables were tested using the Chi-Squared test (or Fisher’s exact test), and continuous variables were tested using t-tests as applicable. Attitudes concerning EoL were also compared between subjects who supported a terminally ill relative and subjects who did not. Adjustments for multiple comparisons by Bonferroni were applied.

Multiple logistic regression analyses were performed to predict different end-of-life care practices. Variables were introduced into the different analyses based on association in the bivariate analysis (i.e., significance up to a level of *p* = 0.2) and after the negation of multi-collinearity. Variables in the different models were adjusted to the age of the dying patient and were removed by a backward elimination method if they were found to be insignificant at a level of *p* ≤ 0.05.

### Ethical considerations

This study was approved by the Institutional Review Board of the Sheba Medical Center (Approval No. SMC-7384-20 dated 10 March 2021). All participants completed and signed an online version of the informed consent form. All data were collected anonymously.

## Results

### Sample descriptive

Of the 605 participants, nearly 50% (*n* = 297) reported supporting a dying terminally ill relative in the last 3 years. These participants provided a detailed account of their relative’s EoL process. Table 1 shows the characteristics of this sample of 297 participants. These characteristics indicate an equal gender distribution and a diverse representation of the ethnic and religious sub-populations in Israel. The variability of age, income, and education levels allows for effective comparative analysis.

**Table 1:** Socio-demographic characteristics of the study sample of caregivers supporting a dying relative at end-of-life (EoL) (N = 297)

Characteristic	Categories	n (%)
Age group (years)	Mean (± SD)	62.61 (± 7.97)
	50–59	117 (39.4)
	60–69	111 (37.4)
	70 +	69 (23.2)
Gender	Female	127 (42.8)
	Male	170 (57.2)
Education (years)	≤ 12	106 (35.7)
	> 12	191 (64.3)
Income	Much below average	65 (24.8)
	Somewhat below average	49 (18.7)
	Same as average	44 (16.8)
	Somewhat above average	66 (25.2)
	Much above average	38 (14.5)
Ethnicity	Jews	263 (88.6)
	Arabs	34 (11.5)
Religiosity*	Secular	84 (31.9)
	Traditional	119 (45.3)
	Religious and ultra-orthodox	60 (22.8)

*Among Jewish participants, only

### Circumstances of EoL

The accounts of the 297 interviewees who supported a dying relative reveal a varied typology. Table 2 provides the EoL characteristics of the protagonist patients as provided by the family caregivers participating in the study. The list of underlying diseases included malignancy (44%), chronic disease, e.g., diabetes, chronic cardiac, renal, and pulmonary disease (48%), and incapacitating diseases, e.g., stroke, dementia, and age-related frailty (45%). About 20% of the protagonists suffered from multiple comorbidities. Interestingly, in 13% of cases, patients faced death when fully independent. Most patients died in a hospital (~ 60%) or a nursing care institution (11%). Only 5.4% died as hospice inpatients. 23% of the patients examined in this study died in their homes.

**Table 2:** End-of-life (EoL) characteristics of the protagonist patient whose EoL process was accounted for by the family caregiver included in the studied sample (*N* = 297)

Characteristic	Categories	n (%)
Relationship of reporting caregiver to the dying patient	First-degree relative (parent, child, sibling)	186 (62.6)
	Second-degree relative (grandparent, cousin, family relative)	61 (20.5)
	Close friend	32 (10.8)
	Other	18 (6.1)
Age at death (years)	Mean (± SD)	73.23 (± 14.88)
	≤ 50	23 (7.7)
	51–60	46 (15.5)
	61–70	46 (15.5)
	71–80	74 (24.9)
	81+	108 (36.4)
Major morbidities experienced before and during EoL	Chronic diseases—all	107 (36.0)
	Heart disease	49 (16.5)
	Diabetes	38 (12.8)
	Pulmonary disease	33 (11.1)
	Kidney disease	23 (7.7)
	Malignant disease—all	140 (47.1)
	Incapacitating diseases—all	121 (40.7)
	Old age	60 (20.2)
	Dementia	41 (13.8)
	Stroke	32 (10.8)
	Morbidity conditions—all	297 (100)
	Malignant (isolated)	116 (39.06)
	Incapacitating (isolated)	69 (3.23)
	Chronic (isolated)	53 (17.85)
	Malignant + Chronic	9 (3.03)
	Malignant + Incapacitating	6 (2.02)
	Chronic + Incapacitating	36 (12.12)
	Chronic + Incapacitating + Malignant	8 (2.69)
Place of death	Hospital (< month hospitalization)	115 (39.8)
	Hospital (≥ month hospitalization)	56 (19.4)
	Home	68 (23.5)
	Geriatric/nursing care institution	32 (11.1)
	Hospice	16 (5.5)
	Other	2 (0.7)
Dependency	Was not dependent	38 (12.8)
	A few days/weeks	73 (24.6)
	Several months up to a year	86 (29.0)
	One to two years	40 (13.5)
	More than 2 years	60 (20.2)

### Factors affecting utilization of end-of-life care practices

This study focused on three major end-of-life care practices: mechanical ventilation, artificial feeding, and the use of strong analgesics. The participants’ reports indicated that at the end of life, 37% of the patients were subjected to mechanical ventilation and 30% to artificial feeding. Close to 60% of the patients were treated with analgesics.

Table 3 utilization of these end-of-life care practices according to the varied characteristics of the patients and their family caregivers. The Table focuses on variants related to the medical profile of the patient and the socio-demographic profile of the accompanying family member (as a proxy of the profile of the diseased patient).

**Table 3:** Utilization of end-of-life-related care practices (EoLCP) according to the family caregiver or patient characteristics (*N* = 297)

Characteristics	Categories	Mechanical ventilation 105/282 (37.2%)	Artificial feeding 84/276 (30.4%)	Analgesic-induced sedation 129/221 (58.4%)
		n/N (%) *P* value	n/N (%) *P* value	n/N (%) *P* value
Medical characteristics of the patient				
Patients age at death	≤ 60	27/67 (40.3)	20/67 (29.8)	41/54 (75.9)
	61–70	18/44 (40.9)	12/43 (27.9)	24/35 (68.6)
	71–80	26/66 (39.4)	18/65 (27.7)	32/51 (62.7)
	81+	34/105 (32.4)	34/101 (33.7)	32/81 (39.5)
	P value	0.6400	0.8330	0.0001
Morbidity leading to death	Malignancy (isolated)	32/113 (28.3)	26/109 (23.8)	73/92 (79.3)
	Chronic Disease (isolated)	32/51 (62.8)	18/47 (38.3)	18/38 (47.4)
	Incapacitation (isolated)	18/62 (29.0)	22/63 (34.9)	17/50 (34.0)
	Complex morbidity	23/56 (41.4)	18/57 (31.6)	21/41 (51.2)
	P value	0.0002	0.2300	< 0.0001
State of dependence	Non Dependent	16/34 (47.1)	11 /35 (31.4)	16/29 (55.2)
	Dep. ≤1 year	56/152 (36.8)	42 /148 (28.4)	82/126 (65.1)
	Dep. >1 year	33/96 (34.4)	31/93 (33.3)	31/66 (47.0)
	P value	0.4148	0.7110	0.0501
Socio-demographic characteristics of family caregivers				
Education	≤ 12 years	37/100 (37.0)	24/96 (25.0)	44/77 (59.5)
	> 12 years	68/182 (37.4)	60/180 (33.3)	85/147 (57.8)
	P value	0.9519	0.1500	0.8158
Income	Below average	49/109 (45.0)	29/103 (28.2)	49/90 (54.4)
	Same as average	18/41 (43.9)	11/40 (27.5)	17/30 (56.7)
	Above average	29/100 (29.0)	31/101 (30.7)	52/77 (67.5)
	P value	0.0442**	0.8965	0.2109
Ethnicity	Jews	92/251 (36.7)	75/247 (30.4)	109/194 (56.2)
	Arabs	13/31 (41.9)	9/29 (31.4)	20/27 (74.1)
	P value	0.5660	0.9410	0.0773
Religiosity among Jews	Secular	29/79 (36.7)	22/82 (26.8)	40/62 (64.5)
	Traditionalist	38/114 (33.3)	34/112 (30.4)	45/86 (52.3)
	Religious + Ultra-orthodox	25/58 (43.1)	19/53 (35.9)	24/46 (52.2)
	P value	0.4537	0.5383	0.2768

**Non-significant after Bonferroni adjustments

The analysis revealed a variability of effects that could be linked to the medical justification for utilizing specific procedures for specific patients’ conditions. For example, mechanical ventilation was associated with chronic diseases (*p* = 0.0002), and potent analgesics were strongly associated with malignancies (*p* = 0.0001). Utilization of mechanical ventilation was not associated with age, nor was artificial feeding (*p* > 0.05 in both). In contrast, the use of strong analgesics to relieve pain and restlessness was more prevalent among patients diagnosed with malignancy who were relatively younger than others. The association between the state of dependency and utilization of end-of-life care practices was less notable (data not shown).

Interestingly, no statistically significant differences were observed concerning the utilization of end-of-life care practices across socio-demographic characteristics of family caregivers.

In parallel to evaluating end-of-life care practices defined as direct medical interventions, the study examined three care-related EoL decisions: involving the patient’s family caregivers in the EoL process, allowing the patient to die at home, and informing patients of their impending death by the medical team.

In 81% of the reported cases, the family was involved in the EoL process and decision-making. The proportion of patients reported to have died at home amounted to 23%. Only 26% of patients were informed about their approaching death. Table 4 provides a breakdown of the distribution of these EoL-related decisions by family caregiver and patient characteristics. Family involvement in treatment was associated with the level of patient dependency (*p* = 0.0031). The caregiver’s income was another notable effector (*p* = 0.006), with lower income negatively affecting family caregivers’ involvement in EoL decisions.

**Table 4:** Distribution of end-of-life-related care practices (EoLCP) according to the family caregiver or patient characteristics (*N* = 297)

Characteristics	Categories	Family involved in EoL process 242/297 (81.5%)	Patient died at home 68/297 ( 22.9%)	Disclosing upcoming death 61/243 (26.2%)
		n/N (%) *P* value	n/N (%) *P* value	n/N (%) *P* value
Medical characteristics of the patient				
Patients age at death	≤ 60	50/ 69 (72.5)	12/69 (17.4)	20/56 (35.7)
	61–70	37/46 (80.4)	13/46 (28.3)	12/33 (36.4)
	71–80	57/74 (86.4)	18/74 (24.3)	27/59 (27.1)
	81+	91/108 (84.3)	25/108 (23.1)	13/85 (15.3)
	P value	0.1367	0.5675	0.0219**
Morbidity leading to death	Malignancy (isolated)	96/116 (82.8)	32/116 (27.6)	39/91 (42.9)
	Chronic Disease (isolated)	39/53 (73.6)	8/53 (15.1)	6/40 (15.0)
	Incapacitation (isolated)	60/69 (87.0)	14/69 (20.3)	4/57 (7.0)
	Complex morbidity	47/59 (79.7)	14/59 (23.7)	12/45 (26.7)
	P value	0.2810	0.3100	< 0.0001
State of dependence	Non Dependent	24/38 (63.2)	8/38 (21.1)	10/33 (30.3)
	Dep. ≤1 year	138/159 (86.8)	39/159 (24.5)	36/117 (30.8)
	Dep. >1 year	80 /100 (80.0)	21/100 (21.0)	15/83 (18.1)
	P value	0.0031	0.7723	0.1140
Socio-demographic characteristics of family caregivers				
Education	≤ 12 years	85/106 (80.2)	20/106 (18.9)	26/83 (31.3)
	> 12 years	157/191 (82.2)	48/191 (25.2)	35/150 (23.3)
	P value	0.6691	0.2184	0.1839
Income	Below average	82/114 (71.9)	25/114 (21.9)	23/92 (25.0)
	Same as average	40/44 (90.9)	8/44 (18.2)	10/36 (27.8)
	Above average	89/104 (85.6)	28/104 (26.9)	22/81 (27.2)
	P value	0.0060	0.4656	0.9270
Ethnicity	Jews	219/263 (83.3)	61/263 (23.2)	151/263 (27.1)
	Arabs	23/34 (67.6)	7/34 (20.6)	51/34 (19.2)
	P value	0.0273**	0.7336	0.3924
Religiosity among Jews	Secular	71/84 (84.5)	20/84 (23.8)	18/62 (29.0)
	Traditionalist	96/119 (80.7)	26/119 (21.8)	26/94 (27.7)
	Religious + Ultra-orthodox	52/60 (86.7)	15/60 (25.0)	12/51 (23.5)
	P value	0.5576	0.8831	0.7940

**Non-significant after Bonferroni adjustments

Dying at home was not associated with any of the examined characteristics of the patient or the family caregiver. Disclosing upcoming death by the medical teams to the dying patient was positively associated with malignancy (*p* < 0.0001).

In order to assess the impact of the socio-normative attitudes of family caregivers on the implementation of end-of-life care practices, a cross-tabulation of utilization of studies practices between non-supporting and supporting caregivers (at any level of support—partial, somewhat, and fully) was conducted. This analysis is provided in Table 5. The data shows that excluding the use of analgesics and death at home, all other end-of-life care practices were implemented more among patients whose caregivers supported the practice in question, even when adjusted for multiple comparisons.

**Table 5:** Implementation of end-of-life care practices (EoLCPs) according to socio-normative support of said practices by family caregivers (*N* = 297)

EoLCP	n (%)		χ^2^	*p* value*
	Among non-supporting caregivers	Among supporting caregivers		
Mechanical ventilation	65 (32.0%)	40 (50.6%)	8.43	0.0037
Artificial feeding	45 (24.2%)	39 (43.3%)	10.49	0.0012
Analgesic-induced Sedation	40 (58.0%)	89 (58.6%)	0.01	0.9352
Family involvement in EoL decision	42 (68.9%)	200 (84.8%)	8.11	0.0044
Death at home	19 (17.1%)	49 (26.3%)	3.35	0.0671
Disclosing upcoming death	29 (19.2%)	32 (39.0%)	10.80	0.0010

Maximum missing per item = 76

*All significant *p* values remain statistically significant after adjustment for multiple comparisons using Bonferroni correction

Multiple logistic regression models were built to examine which parameters were independently associated with the above-mentioned end-of-life care practices. Overall, as can be seen in Table 6, results from the multiple logistic regression models supported the findings in the bivariate analysis, except for the results for the outcome of disclosing the upcoming death to the patient, in which age group of the dying patient was no longer significantly related in the multiple logistic analysis.

**Table 6:** Final models of the logistic regression analyses predicting support of four end-of-life-related care practices (EoLCP) (*N* = 297)

EOL practice	Variable	Comparison categories	OR	95% CI for OR		*p* value
				Lower	Upper	
(A) Was not connected to ventilation	Age of patient at death					0.1331
		61–70 versus ≤ 60	1.381	0.594	3.211	0.5687
		71–80 versus ≤ 60	1.839	0.814	4.155	0.5766
		81 + versus ≤ 60	2.651	1.159	6.063	0.0353
	Death cause					< 0.0001
		Chronic versus malignant	0.168	0.077	0.366	0.0002
		Combined versus malignant	0.346	0.155	0.774	0.3854
		Old age versus malignant	0.580	0.257	1.311	0.2285
(B) Was not artificially fed	Age of patient at death					0.6013
		61–70 versus ≤ 60	1.037	0.436	2.467	0.7726
		71–80 versus ≤ 60	1.113	0.515	2.406	0.5363
		81 + versus ≤ 60	0.722	0.363	1.438	0.1843
	Normative belief	Disagree versus agree	2.53	1.469	4.376	0.0008
(C) Received analgesics that could induce sedation	Age of patient at death					0.3162
		61–70 versus ≤ 60	1.010	0.364	2.802	0.6377
		71–80 versus ≤ 60	1.042	0.393	2.763	0.5124
		81+ versus ≤ 60	0.547	0.210	1.422	0.0722
	Death cause					0.0022
		Chronic versus malignant	0.280	0.117	0.674	0.3260
		Combined versus malignant	0.351	0.139	0.889	0.8691
		Old Age versus malignant	0.187	0.075	0.462	0.0131
(D) Family caregivers were involved in EoL care	Age of patient at death					0.6208
		61–70 versus ≤ 60	1.582	0.569	4.399	0.7410
		71–80 versus ≤ 60	1.898	0.703	5.124	0.3621
		81 + versus ≤ 60	1.333	0.549	3.240	0.8318
	Dependency status					0.0034
		More than one year before death versus dependent less than one year before death	0.407	0.188	0.884	0.7121
		Not dependent versus dependent less than one year before death	0.218	0.086	0.552	0.0126
	Level of education	Academic versus not academic	0.327	0.152	0.705	0.0043
	Level of income					0.0089
		Average versus more than average	2.958	0.909	9.624	0.4143
		Less than average versus more than average	3.359	1.463	7.711	0.1414
	Normative belief	Disagree versus agree	0.306	0.141	0.665	0.0028

Variables were introduced into the regression analysis only if they were found to be associated with each dependent EoLCP in the bivariate analysis (*p* ≤ 0.2). The table shows the final model, which includes only variables that maintained their statistical significance up to a level of *p* = 0.05 after adjusting for the patient’s age at death

## Discussion

This study aimed to provide a typology of end-of-life care practice utilization in Israel and its associated factors. In addition, the study sought to account for the extent to which the utilization of these practices in Israel adheres to key principles, namely being medically appropriate per the patient’s status, respecting the patient’s autonomy, and the just utilization of these practices without prejudice. Of note, the present paper is the last in a set of five publications addressing various aspects of end-of-life in Israel. The first one, published by Velan et al. (2019) [11], addresses the attitude of Israeli physicians towards life termination and truth-telling to terminally ill patients. The second one addresses the attitude of the Israeli population toward the same issues [12]. The third one by Tawil et al. (2023) examines qualitatively the role of family caregivers during the EoL process [15]. The fourth one by Bodas et al. (2023) relates to the divergent attitude of the divided Israeli population to health measures at the end-of-life [16], and the current one examines the actual implementations of such practices. Taken together, these papers provide a comprehensive picture of how end-of-life is perceived and practiced in Israel.

### Equity in the utilization of end-of-life care practices

The findings of this study shed important light on several aspects of EoL processes observed in Israel. First, the similar implementation of end-of-life care practices across socio-demographic variables suggests that patients are treated equally once they enter the health system as terminally ill patients at the end of life. Similar findings were reported for the health system in Israel for other types of patients, for example, trauma casualties [17, 18].

Inequities faced by historically underserved populations at the end of life are often a continuation of inequities faced throughout their life. Some of the most common factors driving health disparities include inaccessible socioeconomic resources, patient-provider ethnicity discordance, and gaps in cultural competency [19]. In Israel, these disparities are often manifested in Arab and ultra-orthodox, immigrant, elderly, and low-income populations, as well as those living in the geographical periphery of the country and those with a lower level of education [20–22]. This often translates to higher morbidity and a lower life expectancy [21, 22].

In this study, we focused on end-of-life care practices aimed at enabling a peaceful death. Arguably, a “good” death is provided by protecting patients from aggressive procedures (mechanical ventilation, artificial feeding) incompatible with their goals of care, by providing effective pain management, and by enabling a supportive environment (e.g., family involvement, dying at home, trustworthy caregivers) [23]. The results of this study suggest that, at least for the variables examined in this study, the healthcare system provides equity in the provision of non-aggressive care. Neither the education level nor income of the family caregiver appears to affect the utilization rates of mechanical ventilation or artificial feeding. The same is true for the administration of analgesia. Similarly, socio-demographic variables did not affect death rates at home or the wish for truth-telling. Moreover, people from presumably underserved groups, such as Arabs or religious Jews, had an equal prospect of aggressive treatment or pain management compared to secular Jewish individuals.

The image of equity in the utilization of end-of-life care practices in Israel is different from that emerging in other places around the world. For example, the utilization of such practices by African Americans in the United States of America is reported as lower compared to other ethnicities [24, 25]. Similar data have been reported in Canada [26] and Australia [27]. Nevertheless, it is important to note that the current study’s findings do not suggest equitable access to medical care in Israel. Instead, patients are treated equitably once they are within hospitals and healthcare facilities.

### Caregivers’ socio-normative attitudes largely determine the EoL process

The second interesting finding of this study is the high involvement of family caregivers in EoL processes in Israel. Furthermore, the data suggest that this involvement is highly dependent on the socio-normative profile of the family caregivers.

Family involvement in care for dying patients can depend on several factors. A significant contributor is the sense of Familism or the centrality of the family [28]. Familism consists of familial obligations, perceived support, and perceiving family as referents [29]. High levels of Familism and social support may predict higher involvement in the care of a family member [28, 30], even if this involves facilitating his death. In particular, it is noteworthy that familism (i.e., the centrality of the family) in Israel remains an identifying mark of Israeli society [31]. Other societies demonstrate varying degrees of familism that may affect the perception of the caregivers’ role at EoL [29, 30, 32, 33].

Involvement in end-of-life care was notably lower among Arab families and families with lower incomes. However, associating degrees of Familism to the variability observed in this study is less plausible. There is no basis for assuming that less affluent families will express lower Familism, and there is no reason to believe that Arab families are less interconnected. On the contrary, studies show that traditional populations, such as the Arab population, tend to exhibit tighter family interactions [31].

The difference observed in the utilization of end-of-life care practices in this study between Israeli Arabs and Jews is more likely to stem from variations in the assertiveness and self-confidence of family caregivers towards the health system. Being involved in the medical decision process is not trivial for lay family members and requires certain boldness and self-assurance. Arguably, these can be more prevalent among the dominant, assertive majority of Israeli Jews, who are known for their *Chutzpah* (audacity), than among the Israeli Arab minority, which may manifest behaviors of a subdued minority group. Similarly, the lesser involvement of low-income families in caring for their loved ones may also be related to a lower sense of self-confidence and more mundane factors, such as being preoccupied with their struggle for economic survival [34].

It is noteworthy that despite the overarching consensus on the importance of family caregivers’ involvement in EoL processes, the current legal situation in Israel does not provide a proper framework for such participation, as family caregivers are not automatic substitute decision-makers when the patient loses decision-making capacity. The Patient’s Right Law (1996) and the Dying Patient Law (2005) are not fully compatible with the findings of this and previous studies [12]. Despite the vacuum created in the absence of such a legal framework, it appears that traditions and habits have developed, allowing family caregivers to impact end-of-life care practices. This is exemplified in our study by the correlation between the socio-normative attitudes of the caregivers and the actual utilization of such practices. In other words, medical teams seem to lend a listening ear to families and, by doing so, provide them with much-needed support [35]. To fully understand the required support caregivers need, future research should look into the extent to which caregivers were treated respectfully by the healthcare providers, the extent to which the patient and the caregivers were offered and provided with emotional support, did the caregivers felt lonely in the EoL process of their loved one, etc. [36–42]. .

### Limitation

This study has several limitations. First, using an online panel to collect data may limit the conclusions to people with high digital literacy. Additionally, online panels in Israel are somewhat limited in their capacity to generate high-quality representative samples of minorities, namely Arabs and ultra-orthodox. While the participation of participants from these sub-populations in the study allows for comparisons, the generalization of the conclusions to these groups should be done with caution. Nevertheless, given the majority of internet users in Israel and the need to administer a multitude of questions to a large sample in a wide geographical distribution, the choice of online sampling was deemed appropriate. Second, this study utilized a sample of participants aged 50 and above; therefore, the conclusions cannot be generalized beyond this age group. The choice to include individuals aged 50 and above was in favor of obtaining a large enough sample of people who accompanied a loved one to their death from a terminal illness. Third, some important questions concerning EoL care remain unanswered. In particular, this study does not account for COVID-19-related deaths, how close the accounting relatives were to the protagonist patient, how close the support they were getting was, what the intensity and frequency of involvement of caregivers were, etc. Lastly, as with all cross-sectional studies, this study is true to its time. Sampling in future dates may yield other patterns in public attitudes. Therefore, following up on this study and assessing changes in a longitudinal study is essential.

### Implications for health policy

End-of-life care is one of the most complex and charged issues faced by medical teams treating terminally ill patients. It is the responsibility of the health establishment to provide guidelines and directives to support clinicians’ and practitioners’ efforts. The implementation of end-of-life care practices is primarily a clinical issue. Yet, health care addressed to a dying person cannot remain a clinical issue per se. It always engenders moral, social, and even political questions. This is especially true for the Israeli context, where end-of-life questions are intermingled with questions related to religion, degree of religiosity, political view, and the tension between modernity and tradition [16]. The findings of the current study are of particular importance to Israeli healthcare practitioners and policymakers, given the growing division in Israeli society [43–45]. One of the major future challenges of health policymakers will be navigating the needs of a highly divisive society. End-of-life issues are good markers for probing the interrelationships between health and schism. This paper provides some source of hope since it demonstrates that flexibility and adaptation can be the solution to overcome future problems.

The study we performed to better understand the dilemmas associated with end-of-life care practices in Israel has shed important light on numerous aspects. It provided a much-needed bridge between the socio-normative attitudes of the Israeli public concerning these practices [12, 15, 16] and the challenges faced by decision-makers. The current study builds upon accumulated insights from this ongoing research and allows the proposition of several implications for policy-making by decision-makers.

The first implication of policy-making pertains to adherence to principles of justice and equity. This study shows that end-of-life care practices and services were provided according to the medical needs, which reinforced the professionalism of the medical teams in Israel. Equity and justice are maintained in spite of the immense social split, suggesting that the strong principles of free-of-cost public services implanted in Israel’s health services have not been hampered. The picture painted by the results of this study proposes that decision-makers are relieved from the pressure of ensuring that these practices in Israel follow the principles of justice and equity, as this seems to be the case already.

The second implication deals with facing the dilemma of responsibility and autonomy in end-of-life care practices. As a modern health system, the Israeli health establishment places value on the autonomy of the patients and their involvement in the medical decision-making of their treatment process. Obviously, the capacity to uphold this standard becomes increasingly difficult as the patient approaches death and under certain medical conditions. It is during these “gray” transition periods where the decision makers’ dilemmas concerning policies become clearer—to what extent can you transition the responsibility over autonomy to the dying patient’s family and caregivers? This current study shows that regarding this issue, end-of-life care practices and services are usually implemented in accordance with the normative beliefs of the patient’s family. The last observation is the most invigorating. In practice, the medical teams adapt themselves to the variable agendas of Israeli subpopulations, distancing themselves from the national debate on end-of-life. These findings suggest that policymakers should seek solutions to seemingly impossible-to-solve problems within the system itself, as it creates these inventive solutions to accommodate personal needs and preferences. Nevertheless, decision-makers cannot shy away from this dilemma, given that the practice by which the family’s wishes are respected is not supported by appropriate legislation and regulation. Policymakers are encouraged to continue a professional discourse surrounding this phenomenon, involving practitioners and public figures, to support a process that will lead, eventually, to the creation of a formal framework to allow family and caregivers to play a legal role in their loved one’s dying process.

The third implication for policy concerns the major rifts in Israeli society concerning attitudes toward end-of-life care practices. Based on this and the previous studies performed by us [11, 12, 15, 16], we can argue that concluding a publicly consented legislation for end-of-life in Israel is an unachievable task. These studies and the current one demonstrate the deep division between the Israeli “tribes” stemming from religious, political, and socio-cultural beliefs as they trickle into EoL decisions and practices. Arguably, any formal attempt to bridge these gaps is doomed to fail. If so, policymakers are urged to consider an alternative approach by which every sector of Israeli society will be allowed to exercise its EoL beliefs. This study shows that de facto, this is already the case. In this regard, perhaps non-policy is the right policy after all.

Lastly, although made with caution, given the limitations in generalizing the results beyond the Israeli context, a couple of global implications for practitioners may be drawn from this study. First, in the socio-cultural context in which familism is pronounced, allowing families to be more involved in end-of-life decisions is justified. Family caregivers would like to be involved in decision-making at the end of their loved one’s life and to reflect their socio-normative beliefs onto this process. End-of-life care practitioners and clinicians can consider allowing interested family caregivers to influence end-of-life care practices with socio-normative attitudes to support them in the process. Second, some of these practices may be more frequent than others. For example, the data of the current study show that close to 60% of caregivers reported that their dying relatives received analgesic treatment that could induce sedation; most of them are young and suffering from malignancies. On the other hand, most patients were not placed on artificial feeding or ventilation. However, these findings should be treated with caution since most caregivers are not health professionals and might report that their loved ones have been subjected to a certain end-of-life care practice, although this was not necessarily the case. A careful analysis of the circumstances under which these practices are being utilized is warranted in each socio-cultural context.

## Conclusions

The findings portray an important image of equity in the utilization of EoLCP in Israel, indicating that once entered into the health system, dying patients receive equal treatment that follows their medical status, regardless of socio-demographic background. Additionally, the findings suggest that family caregivers’ involvement is highly dependent on their socio-normative profile and that medical teams adapt to family caregivers’ wishes accordingly. Despite the overarching consensus on the importance of family caregivers’ involvement in EoL processes, the current legal situation in Israel does not provide a proper framework for such participation. Health system leaders should resist the call for legislation that would impose uniform end-of-life care practices for all Israelis. Instead, they should take steps to preserve and enhance the widespread current practice of practitioners to adapt EoL care to the varied needs and preferences of Israeli families and cultural/social/religious subgroups. These steps should include providing frameworks and tools for family caregivers to support their loved ones close to their deaths, such as educational programs, seminars, supportive care before and during the end of life of their loved ones, etc.

## Data Availability

The datasets used and/or analysed during the current study are available from the corresponding author upon reasonable request.
